# A Five-Gene Signature for Recurrence Prediction of Hepatocellular Carcinoma Patients

**DOI:** 10.1155/2020/4037639

**Published:** 2020-10-24

**Authors:** Zeyu Wang, Ningning Zhang, Jiayu Lv, Cuihua Ma, Jie Gu, Yawei Du, Yibo Qiu, Zhiguang Zhang, Man Li, Yong Jiang, Jianqiu Zhao, Huiqin Du, Zhiwei Zhang, Wei Lu, Yan Zhang

**Affiliations:** ^1^Liver Cancer Center, Tianjin Medical University Cancer Institute and Hospital, National Clinical Research Center for Caner, Key Laboratory of Cancer Prevention and Therapy, Tianjin's Clinical Research Center for Cancer, Tianjin 300060, China; ^2^Department of Liver Transplantation, Tianjin First Central Hospital, Tianjin 300192, China; ^3^Post-Doctoral Research Center, Nankai University, Tianjin 300071, China; ^4^Department of Hepatobiliary Surgery, the First Central Clinical College of Tianjin Medical University, Tianjin 300192, China; ^5^Department of Gastroenterology, the Second Hospital of Tianjin Medical University, Tianjin 300211, China; ^6^Department of Cardiology, the Second Hospital of Tianjin Medical University, Tianjin 300211, China; ^7^Department of Gastroenterology, Tianjin Haihe Hospital, Tianjin 300350, China

## Abstract

**Background:**

Hepatocellular carcinoma (HCC) is one of the most aggressive malignancies with poor prognosis. There are many selectable treatments with good prognosis in Barcelona Clinic Liver Cancer- (BCLC-) 0, A, and B HCC patients, but the most crucial factor affecting survival is the high recurrence rate after treatments. Therefore, it is of great significance to predict the recurrence of BCLC-0, BCLC-A, and BCLC-B HCC patients.

**Aim:**

To develop a gene signature to enhance the prediction of recurrence among HCC patients.

**Materials and Methods:**

The RNA expression data and clinical data of HCC patients were obtained from the Gene Expression Omnibus (GEO) database. Univariate Cox regression analysis and least absolute shrinkage and selection operator (LASSO) regression analysis were conducted to screen primarily prognostic biomarkers in GSE14520. Multivariate Cox regression analysis was introduced to verify the prognostic role of these genes. Ultimately, 5 genes were demonstrated to be related with the recurrence of HCC patients and a gene signature was established. GSE76427 was adopted to further verify the accuracy of gene signature. Subsequently, a nomogram based on gene signature was performed to predict recurrence. Gene functional enrichment analysis was conducted to investigate the potential biological processes and pathways.

**Results:**

We identified a five-gene signature which performs a powerful predictive ability in HCC patients. In the training set of GSE14520, area under the curve (AUC) for the five-gene predictive signature of 1, 2, and 3 years were 0.813, 0.786, and 0.766. Then, the relative operating characteristic (ROC) curves of five-gene predictive signature were verified in the GSE14520 validation set, the whole GSE14520, and GSE76427, showed good performance. A nomogram comprising the five-gene signature was built so as to show a good accuracy for predicting recurrence-free survival of HCC patients.

**Conclusion:**

The novel five-gene signature showed potential feasibility of recurrence prediction for early-stage HCC.

## 1. Introduction

Hepatocellular carcinoma (HCC) is the sixth most frequent malignancies worldwide with contributing to the third population of cancer-related deaths [1]. Barcelona Clinic Liver Cancer (BCLC) staging system is based on liver function and tumor status. It has been adopted worldwide as an crucial staging method for judging patient's prognosis and guiding clinical treatment choices [2]. BCLC-0 and BCLC-A HCC patients can receive curative treatments which achieve good prognosis. Although transarterial chemoembolization (TACE) is recommended as the standard treatment for BCLC-B HCC, increasing researches indicating treatment strategies including liver resection provided a survival benefit over TACE in BCLC-B HCC [3–9]. However, tumor recurrence after surgery is a challenging clinical problem [10]. The cumulative 5-year recurrence rate reported from centers in the east and the west was in the range of 60% to 100% [11]. After liver transplantation, the 5-year recurrence rate is estimated at between 5% and 15% in the literature [12].

At present, the treatment strategies of HCC are mostly based on staging and clinical characteristics, lacking molecular markers for accurate assessment of recurrence and prognosis. Few researches focusing on recurrence models of the early or intermediate stage of HCC patients [13]. Some studies discovered that related genes are related to HCC recurrence and can be introduced as predictive markers. Ren et al. reported that the deregulation of CYP2A6 and CYP2C8 is linked to worse recurrence-free survival (RFS) for HCC [14]. A study conducted by Yang et al. demonstrated that overexpression of APOBEC3F in tumor tissues is potentially predicting the poor recurrence-free survival of HBV-related HCC [15]. Considering the limitation predictive value of single mRNA, some researches were focused on the integrated model composed of multiple genes. Gu et al. conducted a six-long noncoding RNA signature (including gene MSC-AS1, POLR2J4, EIF3J-AS1, SERHL, RMST, and PVT1) to predict the recurrence-free survival of HCC [16]. Besides, the prognostic value of the gene signature to predict recurrence of HCC patients has not been fully illustrated. More molecular markers and models are needed to be developed to assist the recurrence prediction.

Hence, we queried the GEO database to screen out molecules related to the prognosis of HCC and establish models as a molecular method for predicting the recurrence of early-stage HCC.

## 2. Materials and Methods

### 2.1. Data Collection

The mRNA expression data and corresponding clinical data were collected from the Gene Expression Omnibus (GEO) database (https://www.ncbi.nlm.nih.gov/geo) till November of 2019. The dataset GSE14520 based on GPL3921 platform (Affymetrix HT Human Genome U133A Array) contained 225 HCC tumor specimens. The tumor specimens with incomplete gene expression information were excluded. The dataset GSE76427 contained 115 human tumor samples which was based on the GPL10558 platform (Illumina HumanHT-12 V4.0 expression beadchip). If duplicate genes were included, the average value of the gene expression was taken.

### 2.2. Differentially Expressed mRNAs in HCC

The data obtained from GSE14520 profile contained the mRNA expression of 21248 genes. Afterwards, the limma package of R software was adopted to screen the differentially expressed mRNAs (DEMs). False discovery rate (FDR) was introduced for testing *P* values. The DEGs were selected with the cut-off criteria adjusted ∣fold change | >1 and *P* value < 0.05. In all datasets, the duplicate genes were taken the average value of the gene expression. The negative number of gene expression in GSE76427 were deleted. Then, we removed the genes which were not included in GSE76427. After the abovementioned ways, the remaining genes were used for further analyses.

### 2.3. Construction of Gene-Related Prognostic Signature

Univariate Cox proportional hazard regression analysis was introduced to assess the relationship between the expression of DEG and recurrence-free survival time of patients with HCC. Then, prognostic mRNAs were further filtered by means of the least absolute shrinkage and selection operator (LASSO) method. Multivariate Cox analysis was conducted to test the independent prognostic factors of RFS. The content of risk score formula was illustrated as follows: (coef of mRNA1 × expression of mRNA1) + (coef of mRNA2 × expression of mRNA2) + (coef of mRNA3 × expression of mRNA3) + ⋯+(coef of mRNAn × expression of mRNAn). The R package ‘survival ROC' was used to draw the time-dependent relative operating characteristic (ROC) curve. Patients were divided into the low-risk and high-risk groups by the cut-off value of the median risk score. The Kaplan-Meier survival curve was used to compare the RFS of the low-risk with high-risk groups.

### 2.4. Validation of the Prognostic Value of the Five-Gene Signature

For the purpose of verifying the expression patterns of the predictive signature, we detected the expression levels of five genes of GSE14520 validation set, the whole GSE14520, and finally in GSE76427. The risk score of each included patient was carefully calculated. Then, we tested the predictive value of five-gene signature by methodology of the Kaplan-Meier and ROC curves. Finally, the patients were divided into the early recurrence (≤1 year) and late recurrence (>1 year) groups. Kaplan-Meier analysis was performed to assess the predictive value of gene signature on the early and late recurrence in GSE14520 and GSE76427.

### 2.5. Building a Predictive Nomogram

We adopted multivariate Cox regression coefficients of clinical characteristics to establish a nomogram to predict the recurrence at critical point of 1 year, 2 years, and 3 years in HCC patients. The concordance index (C-index) was used to assess the predictive accuracy of the nomogram. The calibration plot was used to verify the probabilities of nomogram prediction. The time-dependent ROC curves of the nomogram model were calculated at the critical point of 1 year, 2 years, and 3 years. Then, the predictive value of the nomogram was verified in GSE76427.

### 2.6. Functional Enrichment Analysis

For the purpose of identifying the molecular mechanisms and pathways of mRNA-based signature, we conducted the Gene Set Enrichment Analysis by performing the Kyoto Encyclopedia of Genes and Genomes (KEGG) pathway and Gene Ontology (GO) analyses using the DAVID (https://david.ncifcrf.gov) online tool. R software was used to display the results of KEGG analyses.

### 2.7. Statistical Analysis

All data were analyzed by R software v3.61 (R Foundation for Statistical Computing, Vienna, Austria). The LASSO Cox regression was undertaken by the ‘glmnet' package. Survival ROC was plotted using the ‘survival ROC' package. The nomogram and calibration plots were conducted by the ‘rms' package. Unsupervised clustering analysis was carried out using the ‘cluster' package. Univariate and multivariate Cox regression analyses were performed to evaluate survival using the ‘survival' package. A *P* value < 0.05 was considered statistically significant in this study.

## 3. Results

### 3.1. Patient Characteristics

The GEO database was thoroughly queried for datasets involving studies of hepatocellular carcinoma. Two datasets, namely, GSE14520 and GSE76427, were selected containing the recurrence-free survival data and the BCLC classification. GSE14520 contains 445 specimen, including 225 HCC specimens and 220 normal tissues. Among the 225 HCC patients, 190 patients with BCLC-0, BCLC-A, and BCLC-B stages were included in the study. All patients were provided with complete clinical and genetic information. Among the 190 patients, the number of BCLC-0, BCLC-A, and BCLC-B patients were 20, 148, and 22, separately. The complete gene expression and clinical data (gender, age, and alanine aminotransferase (ALT)), cirrhosis, main tumor size, multinodular, BCLC stage, tumor node metastasis (TNM) stage, and *α*-fetoprotein (AFP) were contained among 185 patients in this dataset. There was a preponderance of male patients (158, 85.41%); the median age was 51 years (range 21-77 years); and 169 patients (91.35%) were diagnosed with cirrhosis, 108 (58.38%) patients with high AFP (>300 ng/ml), 114 (61.62%) patients with high ALT (>50 U/l), and 53 patients (28.65%) with active viral replication chronic carrier (AVR-CC) (Table 1). GSE76427 contain 115 tumor tissues and 52 normal tissues. Among them, 100 patients were with complete clinical information (BCLC classification and RFS information). These patients were included in the GSE76427 validation dataset.

### 3.2. Selection of Differentially Expressed mRNAs

**Figure 1:**
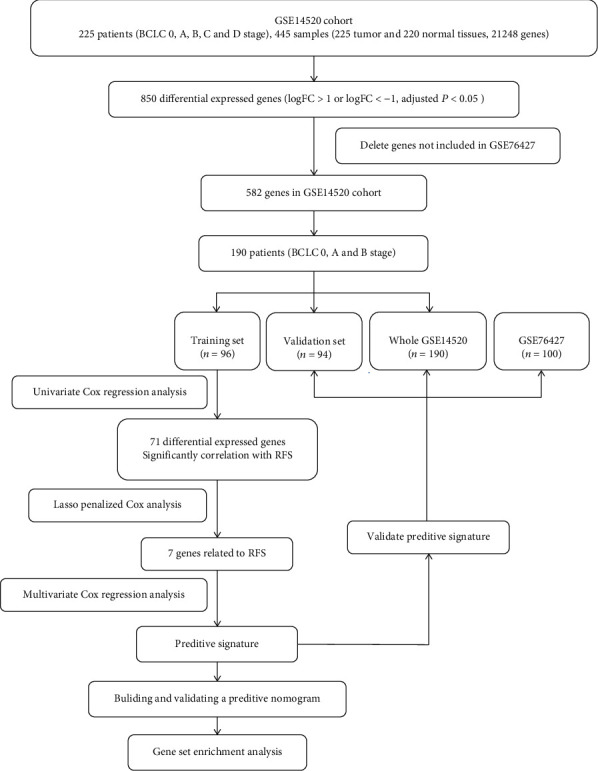
The procedure flow chart of our study on mRNA predictive signature. Abbreviations. HCC: hepatocellular carcinoma; LASSO: least absolute shrinkage and selection operator; ROC: receiver operating characteristic; GEO: Gene Expression Omnibus.

A total of 21248 genes were contained in GSE14520. Compared with normal ones (*n* = 220), 850 genes were found differentially expressed in tumor tissues (logFC > 1 or logFC < −1, adjusted *P* < 0.05). 268 genes not included in GSE76427 were removed. 582 genes remained for further analysis. Among them, 282 mRNAs were upregulated, while 300 genes were downregulated. A flow chart of analysis process was performed to better indicate our research (Figure 1).

### 3.3. Construction of the Prognostic Signature

**Figure 2:**
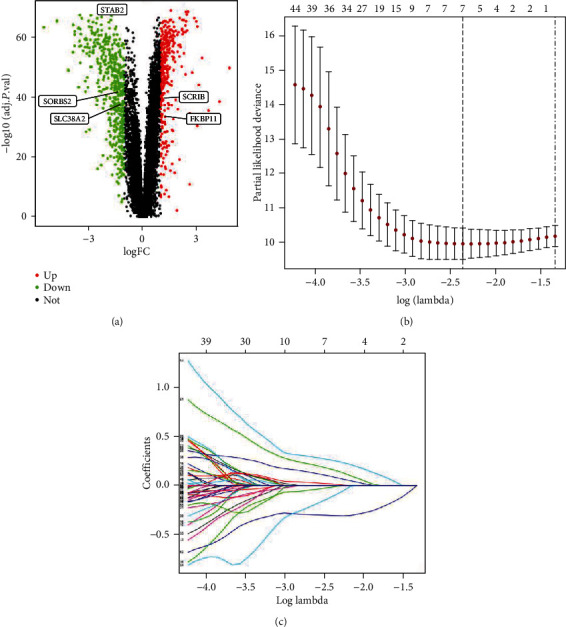
Identification of prognostic genes in HCC patients. (a) Volcano plot of differentially expressed mRNAs. (b) The LASSO model. (c) Plots of the cross-validation error rates. Abbreviations. HCC: hepatocellular carcinoma; LASSO: least absolute shrinkage and selection operator.

**Figure 3:**
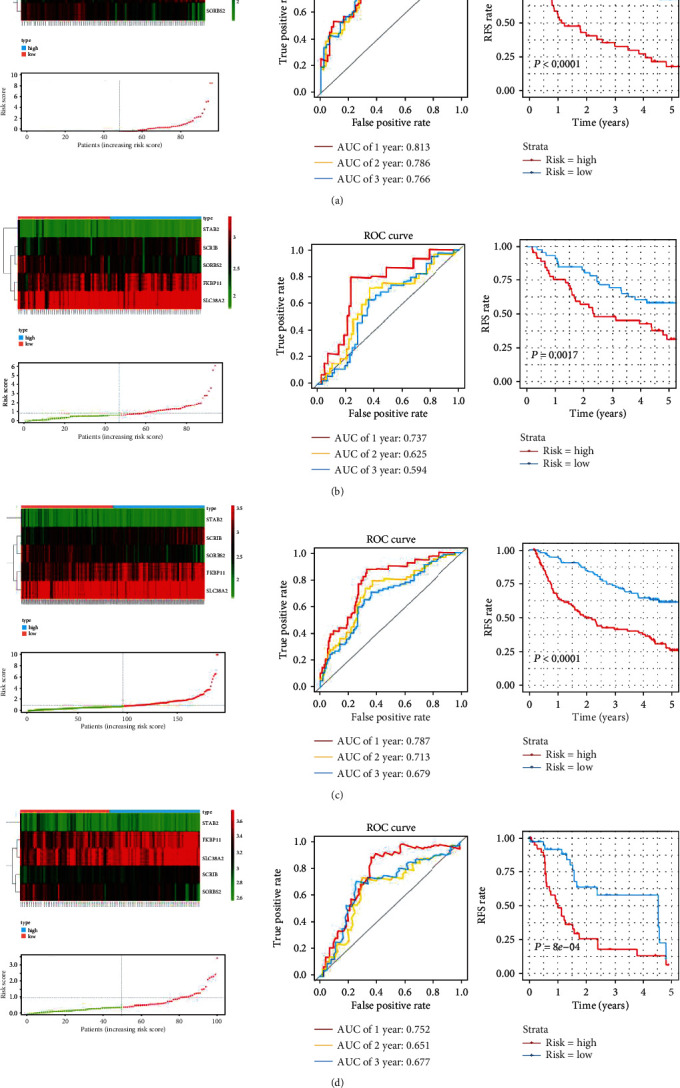
Heatmap of mRNA expression, risk score, time-dependent ROC curve, and Kaplan-Meier survival curve for signature. (a) Heatmap of mRNA expression, risk score, time-dependent ROC curve, and Kaplan-Meier survival curve in the training set of GSE14520. (b) Heatmap of mRNA expression, risk score, time-dependent ROC curve, and Kaplan-Meier survival curve in the validation set of GSE14520. (c) Heatmap of mRNA expression, risk score, time-dependent ROC curve, and Kaplan-Meier survival curve in the whole GSE14520. (d) Heatmap of mRNA expression, risk score, time-dependent ROC curve, and Kaplan-Meier survival curve in the GSE76427. Abbreviations. HCC: hepatocellular carcinoma; ROC: receiver operating characteristic.

A total of 190 patients from GSE14520 were randomly separated into a training group (*n* = 96) and a test group (*n* = 94). To identify the genes with prognostic values, the univariate Cox regression analysis was applied using the ‘survival' package in the training set. The univariate Cox regression identified 71 genes which were associated with RFS. The glmnet package was fully utilized to perform the LASSO Cox regression analysis. Filtered by LASSO analysis (Figures 2(b) and 2(c)), 7 genes remained for further analysis. The risk score was generated by each patient through calculating the values of selected genes which were weighted by their corresponding coefficients in the multivariate Cox regression analysis. After multivariate Cox regression analysis, 5 genes (FKBP11, SCRIB, SLC38A2, SORBS2, and STAB2) were selected to build the predictive signature (Figure 2(a)). Based on these five genes, the biomarker signature which was the calculated risk score shall be listed as follows: risk score = 0.256 × FKBP11 + 0.459 × SCRIB + 0.446 × SLC38A2 − 0.343 × SORBS2 − 0.736 × STAB2. Basing on the risk score signature, the five-gene expression risk score of the 96 patients was calculated separately. Patients were further stratified into the high-risk group (48 patients with 50%) and low-risk group (48 patients with 50%). Patients with lower risk scores were of the characteristics of better RFS. The AUCs of the time-dependent ROC curve were 0.813, 0.786, and 0.766 for 1-, 2-, and 3-year RFS in the training set. The survival analysis demonstrated the result that patients in a high-risk group had significantly poorer RFS than low-risk group patients (*P* < 0.001, Figure 3(a)). These results demonstrated the predictive value of this prognostic signature for recurrence.

### 3.4. Validation of the Prognostic Signature

To verify the accuracy of the five-gene signature in different populations, the patient's risk score was calculated for patients in the GSE14520 testing set. The results showed the RFS of patients in the high-risk group was significantly lower than the patients of the low-risk group (*P* = 0.0017). The area under the curves (AUC)s of the ROC curve were 0.737 at 1-year, 0.625 at 2-year, and 0.594 at 3-year RFS rates (Figure 3(b)). Taken together, the prognostic accuracy of the five-gene signature in the whole GSE14520 was also evaluated with the AUC 0.787, 0.713, and 0.679 at 1, 2, and 3 years, separately. The RFS of patients in the high-risk group was significantly lower than the low-risk group (*P* < 0.0001; Figure 3(c)). The GEO dataset of GSE76427 was applied to further demonstrate the predictive value of the five-gene signature. The AUCs of the ROC curve were 0.752 at 1-year, 0.651 at 2-year, and 0.677 at 3-year RFS. The Kaplan-Meier curve introduced a significantly worse RFS result in the high-risk group than the ones in the low-risk group in GSE76427 (*P* < 0.001; Figure 3(d)). The Kaplan-Meier analysis demonstrated that the predictive value of the gene signature showed good performance in early recurrence (*P* < 0.001) and late recurrence patients (*P* = 0.015) in GSE14520. Patients in the high-risk group had significantly poorer RFS than the low-risk group patients in early recurrence patients of GSE76427 (*P* < 0.001). However, there were insignificantly differences between the high-risk group and low-risk group in the late recurrence of GSE76427 (*P* = 0.19).

### 3.5. Independent Recurrence Prediction of Five-Gene Signature from Other Clinical-Pathological Variables

Univariate and multivariate Cox regression analyses were performed for further test of the five-gene signature independent role of other clinical-pathological variables. The results of the adjustment for conventional clinical patterns by univariate Cox regression analysis, including gender, age, ALT, cirrhosis, main tumor size, multinodular, BCLC stage, TNM stage, CLIP stage, AVR-CC, AFP, and risk score, indicated that TNM stage, BCLC stage, and risk score acted as independent prognostic factors for RFS of HCC patients. Further, multivariate analysis demonstrated that the BCLC stage (HR = 1.926; 95% CI 1.070-3.469; *P* = 0.029) and risk score (HR = 1.313; 95% CI 1.153-1.497; *P* < 0.001) had significant effects on RFS (Table 2).

### 3.6. Recurrence Prediction of Five-Gene Signature in BCLC-0 and BCLC-A HCC

**Figure 4:**
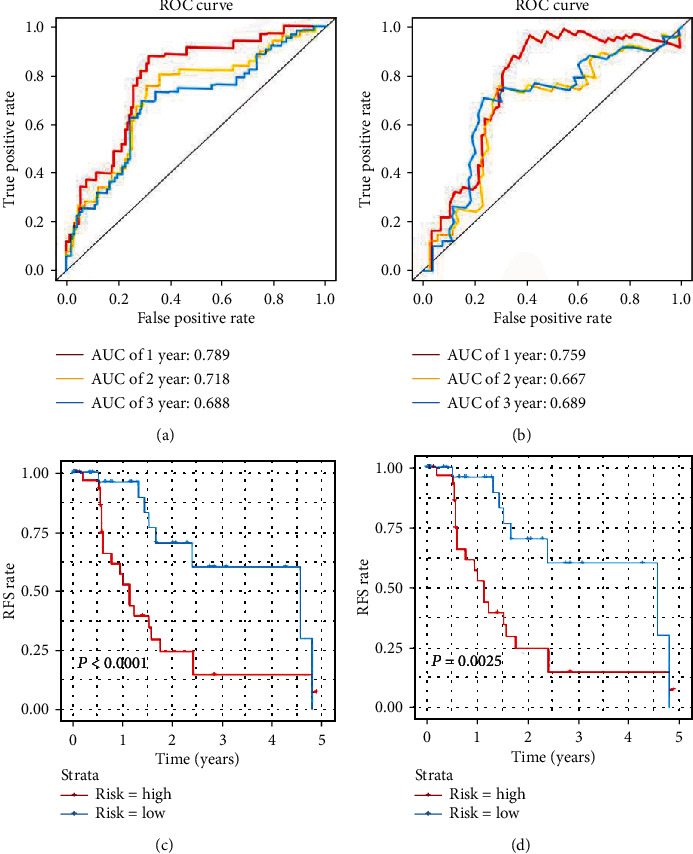
RFS prediction of five-gene signature in BCLC-0 and A HCC. (a) Time-dependent ROC curve of GSE14520. (b) Time-dependent ROC curve of GSE76427. (c) Kaplan-Meier survival curve of GSE14520. (d) Kaplan-Meier survival curve of GSE76427. Abbreviations. HCC: hepatocellular carcinoma; ROC: receiver operating characteristic; RFS: recurrence-free survival.

Due to the curative treatments for patients with BCLC stage 0 or A, the subgroup analysis focusing on the predictive role of gene models of curative treatments for patients with BCLC-0 and BCLC-A was conducted. The patient number of BCLC-0 and BCLC-A in GSE14520 and GSE76427 datasets were 178 and 75, respectively. In GSE14520, the AUCs for 1-, 2-, and 3-year RFS predictions were 0.789, 0.718, and 0.688, respectively (Figure 4(a)). The AUCs for 1-, 2-, and 3-year RFS predictions in GSE76427 were 0.759, 0.667, and 0.689, respectively (Figure 4(b)). The Kaplan-Meier survival curve revealed that the high-risk group patients obtained significantly worse RFS in GSE14520 (*P* < 0.0001), so as in the GSE76427 (*P* = 0.0025) (Figures 4(c) and 4(d)).

### 3.7. Building the Predictive Nomogram

**Figure 5:**
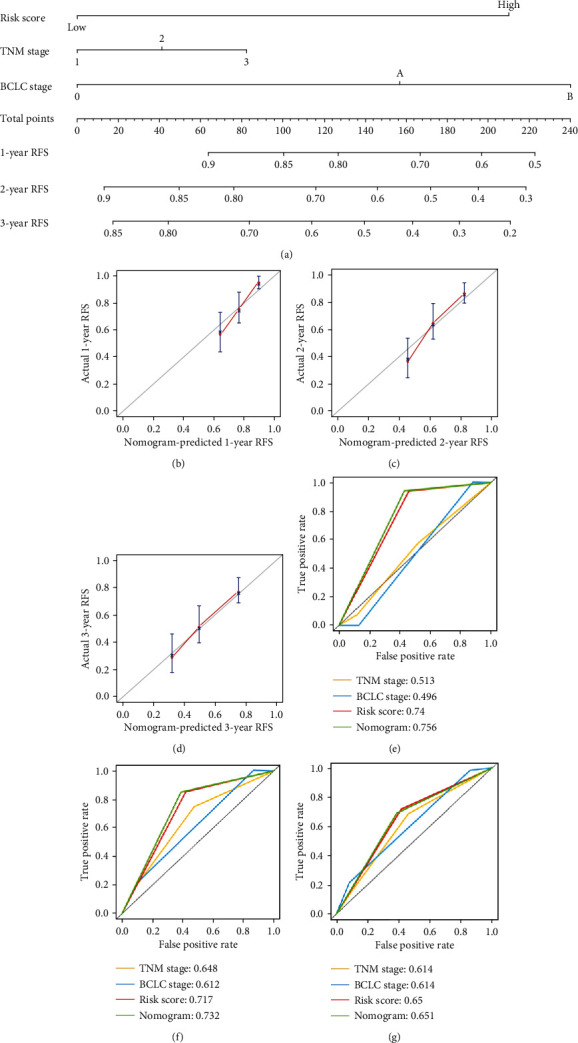
Nomogram model for recurrence prediction. (a) RFS-associated nomogram. (b–d) The calibration curve for predicting 1-year, 2-year, and 3-year, RFS for patients with HCC. (e–g) The time-dependent ROC curves of the nomograms for 1-year, 2-year, and 3-year RFS.Abbreviations. HCC: hepatocellular carcinoma; ROC: receiver operating characteristic; RFS, recurrence-free survival.

Univariate Cox analysis was conducted for clinical factor analysis. To provide the clinician with a quantitative method by which to predict a patient's probability of recurrence, a nomogram that is integrated with three independent prognostic factors (BCLC stage, TNM stage, and risk score; Figure 5(a)) was constructed and developed. The nomogram effectively predicted recurrence rate, with a bootstrapped corrected C-index of 0.684 and AUCs of the ROC curve were 0.756 at 1 year, 0.732 at 2 years, and 0.651 at 3 years, compared with TNM stage, BCLC stage, and risk score, showing the better predictive ability. The C-index value of the nomogram does not contain the five-gene prognostic signature which was much lower than that of combined signature (0.595). The calibration curves for the probability of recurrence at 1 year, 2 years, and 3 years showed good consistency of prediction of nomogram and the actual observations (Figures 5(b)–5(d)). The analysis indicated that compared with the model of TNM stage, BCLC stage, or five-gene signature, the nomogram model obtained more satisfied AUCs for 1-year, 2-year, and 3-year RFS (Figures 5(e)–5(g)). The nomogram was also verified in RFS in GSE76427; AUCs of the ROC curve were 0.735 at 1 year, 0.68 at 2 years, and 0.693 at 3 years showed good performance.

### 3.8. GO and KEGG Pathway Enrichment Analyses

**Figure 6:**
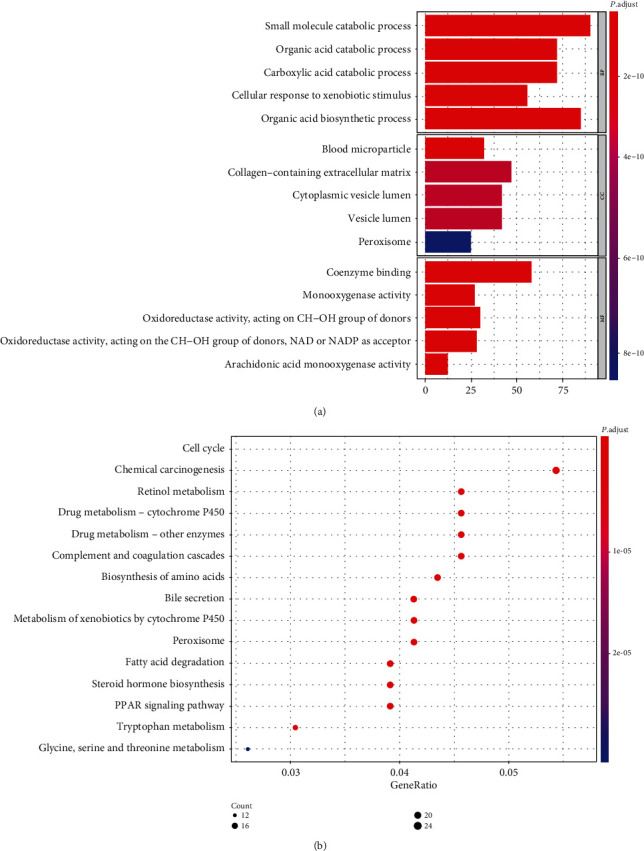
GO and KEGG pathway analyses. (a) Bar graph of gene ontology enriched in molecular function, cellular component, and biological process. (b) KEGG pathway analysis.Abbreviations. KEGG: Kyoto Encyclopedia of Genes and Genomes; GO, Gene Ontology.

To further identify the potential biological functions and mechanisms of these five prognostic mRNAs, Gene Ontology (GO) and Genomes (KEGG) pathway enrichment analysis was performed on the DEGs. For molecular function, DEGs were mainly enriched in coenzyme binding, monooxygenase activity, oxidoreductase activity, acting on CH-OH group of donors, oxidoreductase activity, acting on the CH-OH group of donors, NAD or NADP as accepter and arachidonic acid monooxygenase activity. DEGs in the cellular component category were mainly associated with blood microparticle, collagen-containing extracellular matrix, cytoplasmic vesicle lumen, vesicle lumen, and peroxisome. The top five enriched terms of biological processes were small molecule catabolic process, organic acid catabolic process, carboxylic acid catabolic process, cellular response to xenobiotic stimulus, and organic acid biosynthetic process (Figure 6(a)). The current results showed that the genes were enriched in the cell cycle pathway, chemical carcinogenesis pathway, and retinol metabolism pathway (Figure 6(b)).

## 4. Discussion

HCC is the sixth most frequent malignancies worldwide, which contributes to the third population of cancer-related deaths [1]. After therapy, high recurrence rate takes up the majority of death among HCC patients [17]. According to the EASL Clinical Practice Guidelines, various treatment options could be applied to patients with BCLC stage 0, BCLC stage A, and BCLC stage B [18]. Patients with BCLC stage 0 may prefer to select liver resection or radiofrequency therapy. The recommended treatments for BCLC stage A patients are liver transplantation, liver resection, or radiofrequency therapy. TACE is the available choice for patients with BCLC stage B [18]. Many current researches have indicated that BCLC stage B patients have better surgical outcomes than TACE [3–9]. Some studies showed similar prognosis that underwent liver resection for patients with BCLC-A and BCLC-B HCC [19]. We therefore enrolled BCLC-B HCC patients in our study for further exploration of the prognosis prediction. The most crucial factor affecting the prognosis of HCC patients is recurrence [20]. Many patients are no longer suitable for recurative treatment when relapse so as to what they may receive is only the systemic or conservative treatments. The prognosis of these patients may be seriously affected. Predicting the recurrence of early-stage HCC is becoming increasingly crucial for the prognosis prediction. Most studies ignored the crucial role of molecular recurrence prediction for the BCLC stage in assisting the treatment decision of HCC, especially in early-stage HCC. We were the pioneer to explore the molecular signature of recurrence prediction for HCC patients with BCLC-0, BCLC-A, and BCLC-B HCC patients based on the public database.

In this research, we explored a novel five-gene signature for HCC recurrence prediction. The LASSO penalized regression was used to better analyze the independent variables simultaneously and pick the most influential variable. The predictive value of the signature were validated in GSE14520 validation set and GSE74627; the cross-validation assure the accuracy of our signature. AUCs of 1-year, 2-year, and 3-year RFS in our current results verified the satisfied accuracy of the gene signature, especially in the early recurrence prediction. The RFS rate of patients in the high-risk group was significantly lower than the patients in the low-risk group. Further, Kaplan-Meier analysis showed that the gene signature has a more important role in early recurrence prediction of HCC, but the role of late recurrence prediction needs further verification. The nomogram integrating multiple predictors we conducted also show a good accuracy for predicting recurrence of HCC patients. KEGG pathway enrichment analysis showed that the DEGs were enriched in the cell cycle pathway, chemical carcinogenesis pathway, and retinol metabolism pathway.

The signature including five genes: FKBP11, SCRIB, SLC38A2, SORBS2, and STAB2. FKBP11 (FK506 binding protein 11) mRNA is abundant in liver tissues. Elevated expression of FKBP11 during the development of HCC and FKBP11 may be an early marker for HCC [21]. SORBS2 (Sorbin and SH3 domain-containing 2) is located in the 4q35 region of the human genome, which participates in the regulation of signal transduction, actin dynamics, and cytoskeleton establishment region of the human genome. It was demonstrated that SORBS2 suppresses HCC metastasis and has the potential to be a novel prognostic marker or therapeutic target in HCC [22, 23]. SCRIB (Scrib) is an evolutionarily conserved component of a common genetic pathway involved in apical-basal cell polarity. Scrib inhibits liver cancer cell proliferation and functioning as a tumor suppressor in liver cancer [24]. SATB2 (special AT-rich sequence-binding protein 2), known as a nuclear matrix-associated transcription factor and epigenetic regulator, is an evolutionarily conserved transcription factor [25]. SLC38A2 is one of the important members in the three SLC families, which transports amino acids as the substrate. No studies have been found on the correlation between these five genes and the prognosis of liver cancer. There are few studies about SATB2- or SLC38A2-related HCC. Our research was the pioneer to explore the recurrence prediction signature of HCC consisting of the 5 genes.

Compared with other clinical indicators (gender, age, ALT, cirrhosis, main tumor size, multinodular, BCLC stage, TNM stage, AFP, and risk score), the BCLC stage, TNM stage, and risk score showed better potential feasibility as independent prognostic factors for the RFS of HCC. Further analysis indicated that compared with the model of the BCLC stage, TNM stage, risk score, and nomogram, the nomogram model obtained the satisfied AUCs for 1-year, 2-year, and 3-year RFS. The prognosis value of BCLC stage 0 or A was separately discussed in the subgroup analysis and showed good performance. Based on the gene signature we have established, patients with a high probability of recurrence after liver resection may add systemic adjuvant therapy such as TACE, sorafenib, or immunotherapy to reduce the possibility of recurrence [26–28]. The patients which showed higher probability of recurrence should be emphasized to shorten the interval of follow-up time after therapy.

Certain limitations still exist in this study. Firstly, the sample size was limited. Though we have worked hard to find as many samples as possible from GEO or other databases, we failed to find more datasets containing recurrence and BCLC stage information. A larger sample size research need to be organized. Secondly, the data we obtained was only from a public database, lacking validation of clinical validation such as race, etiology, microvascular invasion, operation, or other types of treatment. Thirdly, the specimen data from the GEO database was with incomplete information, especially lacking of surgical modality information of HCC patients. Further study needs to be explored to focus on verifying the prognostic signature with clinical specimens.

## 5. Conclusions

In conclusion, we explored a five-gene predictive signature with the potential feasibility for the prediction of RFS for hepatocellular carcinoma patients. Based on this predictive signature, more accurate predictive signature combined with clinical characteristics and other omics results would be developed, providing individualized treatments for patients with HCC.

## Figures and Tables

**Table 1:** Demographic characteristics of HCC patients.

Clinical and pathological variable	GSE14520 (*n* = 190)	GSE76427 (*n* = 100)
Gender (male/female)	161/29	79/21
Age (years)	51.22 ± 10.75	63.32 ± 12.8
AVR-CC (no/yes/NA)	132/53/5	NA
ALT (<=50 U/l/>50 U/l)	117/73	NA
Main tumor size (<=5 cm/>5 cm/NA)	133/56/1	NA
Multinodular (no/yes)	157/33	NA
Cirrhosis (no/yes)	16/174	NA
TNM stage (I-II/III-IV/NA)	168/22/0	85/14/1
CLIP stage(0/1/2/3)	94/70/25/1	NA
BCLC stage (0/A/B)	20/148/22	4/71/25
AFP (<=300 ng/ml/>300 ng/ml/NA)	108/79/3	NA

Abbreviations: AVR-CC: active viral replication chronic carrier; ALT: alanine aminotransferase; TNM: tumor node metastasis; CLIP: cancer liver Italian program; BCLC: Barcelona Clinic Liver Cancer; AFP: alpha-fetoprotein.

**Table 2:** Univariate and multivariate analyses of risk factors related to RFS in HCC patients.

Clinical factor	Univariate analysis			Multivariate analysis		
	HR	95% CI	*P* value	HR	95% CI	*P* value
Gender (male vs. female)	0.656	0.300-1.440	0.294			
Age	0.999	0.977-1.022	0.940			
ALT (<=50 U/L vs. >50 U/L)	1.022	0.617-1.694	0.931			
Main tumor size(<=5 cm vs. >5 cm)	1.620	0.958-2.740	0.072			
Multinodular (no vs. yes)	1.556	0.871-2.782	0.135			
Cirrhosis (no vs. yes)	3.428	0.838-14.022	0.087			
AVR-CC (no vs. yes)	0.700	0.386-1.269	0.240			
CLIP stage (0 vs. 1-5)	0.848	0.517-1.391	0.514			
TNM stage (I+II vs. III+IV)	1.758	1.242-2.489	0.001	1.359	0.906-2.038	0.138
BCLC stage	2.586	1.545-4.331	<0.001	1.926	1.070-3.469	0.029
AFP (<=300 ng/ml vs. >300 ng/ml)	0.784	0.477-1.287	0.335			
Risk score	1.316	1.161-1.491	<0.001	1.313	1.153-1.497	<0.001

Abbreviations: AVR-CC: active viral replication chronic carrier; ALT: alanine aminotransferase; TNM: tumor node metastasis; CLIP: cancer liver Italian program; BCLC: Barcelona Clinic Liver Cancer; AFP: alpha-fetoprotein.

## Data Availability

The gene expression and clinical data of HCC in our study were obtained from GEO (https://www.ncbi.nlm.nih.gov/geo).

## Abbreviations

HCC: Hepatocellular carcinoma
LASSO: Least absolute shrinkage and selection operator
ROC: Receiver operating characteristic
GEO: Gene Expression Omnibus
RFS: Recurrence-free survival
KEGG: Kyoto Encyclopedia of Genes and Genomes
GO: Gene Ontology
C-index: Concordance index
BCLC: Barcelona Clinic Liver Cancer
AUC: Area under the curve
AVR-CC: Active viral replication chronic carrier
ALT: Alanine aminotransferase
CLIP: Cancer liver Italian program
TNM: Tumor node metastasis
AFP: Alpha-fetoprotein
DEMs: Differentially expressed mRNAs
FDR: False discovery rate
TACE: Transarterial chemoembolization.
